# Emotional self-knowledge profiles and relationships with mental health indicators support value in ‘knowing thyself’

**DOI:** 10.1038/s41598-024-57282-w

**Published:** 2024-04-04

**Authors:** Jacqueline Nonweiler, Jaume Vives, Neus Barrantes-Vidal, Sergi Ballespí

**Affiliations:** 1 Department of Clinical and Health Psychology, Autonomous University of Barcelona, Edifici B, Campus de Bellaterra, Carrer de la Fortuna s/n, Cerdanyola del Vallès, 08193 Barcelona, Spain; 2 Department of Psychobiology and Methodology of Health Sciences, Autonomous University of Barcelona, Edifici N, Campus de Bellaterra, Carrer de la Fortuna s/n, Cerdanyola del Vallès, Barcelona, Spain; 3 CIBER de Salud Mental, Instituto de San Carlos III, Madrid, Spain

**Keywords:** Emotional self-awareness, Self-knowledge, Self-mentalizing, Positive mental health, Adolescence, Psychology, Human behaviour

## Abstract

“Know thyself” may be indicated by a balanced high pairing of two emotional self-knowledge indicators: attention to emotions and emotional clarity. Closely associated but often evaluated separately, *emotional clarity* is consistently, inversely associated with psychopathology, while evidence regarding *attention to emotions* is less consistent. Variables of high/low *emotional clarity* and *attention to emotions* yielded four emotional self-knowledge profiles which were analyzed for associations with mental health indicators (depression and anxiety symptoms, self-esteem, self-schema, resiliency, transcendence) in *n* = 264 adolescents. Here we report regression models which show that compared with *neither*, *both high (*attention + clarity*)* show higher positive self-schema (*B* = 2.83, *p* = 0.004), more resiliency (*B* = 2.76, *p* = 0.015) and higher transcendence (*B* = 82.4, *p* < 0.001), while *high attention only* is associated with lower self-esteem (*B* = − 3.38, *p* < 0.001) and more symptoms (*B* = 5.82, *p* < 0.001 for depression; *B* = 9.37, *p* < 0.001 for anxiety). *High attention only* is associated with most severe impairment all indicators excepting transcendence. Profiles including high clarity suggest protective effects, and ‘implicit’ versus ‘explicit’ emotional awareness are discussed. Balanced vs. imbalanced emotional self-awareness profiles dissimilarly affect mental health, which have implications for treatment and policy.

## Introduction

*“Self-knowledge is temperance, and I am at one with him who put up the inscription of those words at Delphi…the god addresses those who are entering his temple [by saying] to each man who enters, “Be temperate!”* Plato proclaimed in Charmides^1^. This passage demonstrates that in ancient Greece, the inscription of the pronaos of the Temple of Apollo at Delphi to *“Know Thyself,”* or *“Gnóthi seautón”*, advised people of their human condition, warning them to “be temperate”, or to be aware of the limits of their mortal state. Now more than two millennia later, self-knowledge remains crucial, and not only for moderation as a gesture of respect to the state of divinity, but also as a pathway to mental health and wellbeing.

While some degree of awareness of one’s inner workings is promoted in all psychotherapies, it is not clear to what extent or how this contributes to mental health. On one hand, insight increases self-knowledge, which could be advantageous in noticing and predicting future behavior and to make better decisions, particularly in socioemotional contexts^2^. Therapeutic approaches like insight-based psychodynamic treatments, mentalization based treatments (MBT), and third-wave cognitive-behavioral therapies support the awareness of one’s inner issues (i.e., emotions, desires, values, thoughts) as a contributing factor to mental health^3,4^. These therapies assume that self-knowledge contributes to self-regulation by default, and in turn generates more genuine experiences of the self, and thus to increased genuine self-expression and well-being^5^.

Conversely, attuning to traumatic or painful emotions or thoughts can be detrimental, and the mind typically avoids integrating or processing this content consciously as a means to protect us from pain^6^. Additionally, for those who have experienced maltreatment or other forms of trauma, attending to emotions can lead to unproductive symptomatic experiences such as psychological re-experiencing when traumas are activated in daily life. This could be the reason why ‘knowing thyself’ and having insight compete in daily life with another popular maxim for coping with emotional distress: ‘out of sight, out of mind’, which could indicate low attention and/or awareness to painful internal events. Nonetheless, it is unknown how of these two seemingly opposite mechanisms compete (or combine) to help people deal with emotional distress and reach emotional well-being.

The complexity of assessing self-awareness does not help to shed light on which mechanisms or elements of the construct indeed engender positive mental health outcomes. Firstly, self-knowledge is a broad concept and researchers often focus on specific aspects of the capacity to ‘know thyself’. Thus, the ability to be aware of one’s own inner states has been explored from perspectives such as mindfulness^7^, insightfulness, intra-personal intelligence^8^, reflective function^9^, and self-mentalizing^10^. Secondly, a wide breadth of measures is used to evaluate emotional self-knowledge, and includes interviews, various self-reports and qualitative methods. Although interviews and experimental techniques are often considered superior to self-report, this may not always be the case with self-reports regarding emotional awareness due to inherent intimacy and introspection that underlies self-awareness, which could lead respondents to be more honest in a private, reflective setting.

In this context, one prominent and popular attempt to operationalize aspects of self-knowledge is Mayer and Salovey’s conceptualization of emotional self-awareness into factors which include attention to emotions and emotional clarity^11^. While attention to emotions is defined as the frequency with which one notices and pays attention to their emotional world, emotional clarity refers to a deeper understanding of the meanings and implications of said emotions. This distinction between simple attention and deeper clarity could be a key factor in understanding why apparently similar tendencies to have high emotional awareness are associated with disparate mental and emotional outcomes. Thus, it is possible that high attention and high clarity do not contribute to mental health in the same way, or that imbalanced pairings of high attention accompanied by high versus low clarity have different effects.

The Trait Meta-Mood Scale-24 (TMMS) was developed to assess these dimensions^12^, and has been used extensively to analyze the associations emotional self-awareness dimensions and various constructs including mental health. Previous research shows that emotional clarity is often inversely associated with psychopathology^13^. Deficits in emotional clarity have been found to independently be associated with symptoms of depression in adults^14,15^ and in children^16^, but additionally with social anxiety^15^, borderline personality, binge eating and alcohol use^15^. Moreover, self-concept clarity which overlaps partially with emotional clarity has been strongly, inversely linked to both autism^14^ and psychosis spectrum disorders^17^. Evidence regarding emotional clarity points to negative relationships with transdiagnostic symptomatology^18–22^, and its opposite is also supported such that high emotional clarity is directly linked to a broad range of positive adjustment such as adaptive attributional style and wellbeing^16,23,24^. Experts suggest that perceiving and understanding emotions requires less cognitive control and allows for more resources to be directed toward goal-oriented cognition and behavior, while those who must spend greater time and effort managing their emotional experiences may not have resources left ‘in the tank’ to dedicate toward self-actualization^16,23,25^.

Alternatively, findings regarding how attention to emotions contributes to mental health and wellbeing are less consistent. Some studies show that attention to emotions is not associated with mental health^26,27^, while other studies show that attention to emotions increases emotional distress and leads some individuals to become overwhelmed, especially in the presence of anxiety^28^ or in the absence of emotional clarity^19,29^. This suggests a ‘dark side’ of emotional self-awareness^30^, whereby attention to emotions alone is not sufficient to engender positive outcomes or to protect from unfavorable ones. Interestingly, one modest study (*n* = 27) which used experience sampling methodology found that higher attention to emotions predicted worse recovery from major depressive disorder better than both positive and negative affect after 1 year^31^, which hints at a harmful effect of attention to emotions on mental health.

As summarized above, studies regarding these facets of emotional self-awareness reveal important findings regarding their disparate contributions to mental health. Nonetheless, in reality, these factors do not strictly work independently of each other, but rather work dependently or in tandem to provide an understanding of the emotional world^32^. Despite the interrelatedness of attention to emotions and emotional clarity, very few studies have explored how attention and clarity combine to contribute to mental health. Those that do suggest that (a) high emotional attention may be detrimental to mental health unless combined with high emotional clarity^33^, (b) the imbalanced pairing of high attention and low clarity could yield poorer outcomes for emotional regulation^32^, which means that an imbalanced combination of higher attention than clarity may result in overwhelm^34^, and (c) high emotional clarity seems to impact mental health positively independently of whether it is paired with high or low attention.

It is possible that the balanced or imbalanced combination of these two dimensions, instead of their separate study, will shed new light on their contributions to mental health, particularly because they appear to contribute differentially to salutogenesis and depend, at least in part, on each other. Since previous findings suggest that attention and clarity do not always influence mental health in the same direction, this makes interpreting their interaction more complex. For example, the interaction of high attention and low clarity would yield the same result quantitatively (e.g. 30 × 10 = 10 × 30). In this context, a categorical perspective would be more adequate to better understand the effects of different pairings of high versus low attention with high versus low clarity.

Different potential pairings of attention to emotions and emotional clarity yield four possible combinations of emotional awareness: high attention and high clarity, low attention and low clarity, and two imbalanced pairings: high attention but low clarity, and low attention but high clarity. Within these pairings, people with high attention and high emotional clarity show a sort of ‘full insight’ or balanced high self-awareness. This style may be most analogous to “knowing thyself”. On the contrary, people with low attention and low clarity show balanced low self-awareness. This style may be more analogous to a tendency toward ‘out of sight, out of mind’.

High attention with low clarity (further referred to as *high attention only*) may lead to emotional dysregulation and overwhelm^34^, because it combines high attention to emotional reactions along with low capacity to comprehend and ‘metabolize’ them^23,24^. Low attention to emotions paired with high clarity (further referred to as *high clarity only*), by comparison, suggests a sort of implicit or automatic mentalizing whereby some people could achieve emotional clarity with low attentional resources. While some believe that successful achievements with low cognitive resources indicate proficiency^35^, it is not clear whether high clarity paired with high or low attention could better contribute to mental health. Literature regarding this imbalance is scant, and thus does not support a solid hypothesis.

**Figure 1:**
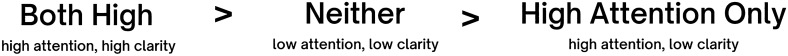
Prediction of favorability of mental health indicators for self-awareness pairings based on previous literature.

The aim of this study is to explore how different combinations of high/low attention to emotions and high/low emotional clarity are associated with various mental health indicators. We encompass research about the contributions of these emotional self-awareness dimensions from a new perspective of analysis, which explores their combined effect. Based on the idea that ‘knowing thyself’ (insight), used as a common factor in most therapies, would be better for salutogenesis than the opposite (‘out of sight, out of mind’) we expect that: (a) those with balanced high attention and high clarity (*both high*; as a sort of ‘full insight’) will show more favorable indicators on positive and negative mental health measures, compared to those with balanced low attention and low clarity (*neither*). Next, based on previous research outlined in detail above, we theorize that (b) those with *high attention only* (high attention but low clarity, thus paying attention to their emotions but not understanding them) demonstrate the poorest outcomes relative to the other groups, as compared to those with *neither* or those with *both high,* those with *high attention only* have awareness of their emotions but lack the clarity to understand, integrate or ‘metabolize’ them. These two hypotheses are graphically summarized in Fig. 1.

Additionally, we are also curious regarding which is more beneficial: high clarity when paired with high attention (‘*both high’*, as a sort of ‘full insight’), or *high clarity only* with low attentional resources (which could indicate proficiency). The lack of previous research prevents us from making solid predictions, but we will additionally explore, with no hypothesis, whether those with *high clarity only* (low attention but high clarity) experience different mental health outcomes than other groups, especially those with *both high* attention and clarity.

## Methods

### Participants

Participants were recruited through secondary schools in Catalonia. They were considered eligible if they were between the ages of 12 and 18 but were excluded if they had a known severe diagnoses such as intellectual disability, psychosis, or autism spectrum disorder. After approaching ten schools with similar characteristics such as urbanicity, size, family socioeconomic status, educational orientation, geographic location, five schools (5/10) agreed to participate and data from *n* = 264 students were included in the study (*n* = 144 biological females, 54.5%). Most of the sample (70.7%) were of a median socioeconomic level (17.7% high; 11.6% low) and approximately 87% were White-European (9% from Arab countries, 2% Asian and 2% Latino).

### Measures

Dimensions of emotional self-awareness (attention and clarity) were measured using the first two dimensions of the Trait Meta-Mood Scale^12^ (attention to emotions and emotional clarity), detailed below. Dimensional measures of depressive and anxious symptoms were used to operationalize distress in the adolescence because of the high prevalence of these symptoms in this stage of life^36^. Because mental health does not refer only to the absence of symptoms or disorders^37^, here we adopt a broader notion of mental health which includes symptoms of common mental illnesses as indicators of distress, but also encapsulates positive aspects such as self-image, resilience, and transcendence (motivation towards life goals). Self-image was operationalized using measures of self-esteem and positive self-schema to encompass both affective and more cognitive aspects of self-image^38,39^. We utilized ego-resilience as an operationalization of resilience, a quality which is defined as an individuals’ ability to flexibly adapt to stressors, both internal and external^40^. Transcendence refers to an orientation toward life goals, and is a measure derived from the Aspiration Index^41^.

#### Emotional self-awareness

Emotional self-awareness was evaluated using one of few measures that exist for evaluating emotional metacognition: the Trait Meta-Mood Scale (TMMS)-24, a shortened version of the original TMMS which includes 48 items^12^. The TMMS-24 includes three factors, attention to emotions, emotional clarity, and emotional repair. The attention to emotions subscale measures attention to feelings, that is, how much attention respondents are paying to their inner feelings and emotions. Emotional clarity evaluates the ability to understand emotions and discriminate between them (Townshend, 2023). For the purposes of this research, attention to emotions and emotional clarity factors were utilized. The TMMS-24 was chosen over the longer version of the same scale for its brevity (given use in a broader project, which included several questionnaires), and the fact that it has experienced more widespread use than the original version. The 24 items (8 per factor) are ranked on a Likert scale from 1 to 5 in accordance with the participant’s level of agreement with each statement. Statements include reflections about one’s own attention to and clarity regarding emotions, such as “I pay a lot of attention to how I feel” and “I almost always know exactly how I am feeling”, respectively. The Spanish version of the TMMS-24 offers good internal consistency with Cronbach’s α range between 0.86 and 0.90, and adequate test–retest reliability (ICC between 0.60 and 0.83)^42^. The current sample further showed excellent internal consistency of this measure (α = 0.90).

#### Symptoms

##### Depression

Perhaps the most widely used and popular measure for depression is the Beck’s Depression Inventory-II (BDI-II)^43^, a self-report measure which was utilized to measure depressive symptoms in the current sample. Each of the 21 items of this sample correspond to a symptom of depression such as sadness, apathy, or suicidal ideation, which are then rated from 0 to 3. The Spanish language version of the BDI shows good psychometric properties in a community sample^44^. Internal consistency for the current sample was α = 0.90.

##### Anxiety

Severity of anxiety was measured using the total score of the Multidimensional Anxiety Scale for Children (30 items, MASC)^45^. This self-report measure includes a total score (the one used in the present study) and four primary subscales: physical symptoms, harm and avoidance, social anxiety, and separation anxiety. Items are ranked from zero to three, which reflects the extent to which each statement applies to the participant (0 = never applies while 3 = often applies). This questionnaire has shown strong reliability and validity in samples that utilize the Spanish version^46^; the internal consistency using Cronbach’s alpha of the current sample was α = 0.88.

#### Resiliency

The Connor Davidson Resilience Scale-10 (CD-RISC10)^47,48^ focuses on an individual’s ability to cope with both internal and external stressors. The version utilized in this research is a shortened version of the original 25-item CD-RISC, which was originally validated in six populations ranging in psychopathology from community sample (no reported psychopathology) to clinical trials for anxiety and post-traumatic stress disorder. The measure reliably distinguishes between individuals with greater and lesser resilience. This shortened measure boasts excellent psychometric properties in all adaptations and includes ten items ranked from 0 to 4 according to the frequency of behaviors such as “able to adapt to change” and “can deal with whatever comes”. The Spanish version of the measure shows excellent internal consistency in a sample of university students (α = 0.85) with a mean score of 27.41 (*SD* = 6.36)^49^. Internal consistency (Cronbach’s alpha) of the measure in the current sample was excellent; α = 0.89.

#### Self-image

##### Self-esteem

Rosenberg’s Self-Esteem Scale (RSES)^50^ is a 10-item self-report measure used to measure self-esteem, the more affective aspect of self-image. Respondents answer the RSES according to a 4-point scale proportional to their level of agreement with statements such as “I feel I do not have much to be proud of” and “I am able to do things as well as most people”. The scale exhibits excellent internal consistency according to the Guttman scale reproducibility coefficient of 0.92. The scale also exhibits excellent stability measured with test–retest reliability intra-class correlations (ICC) of 0.85 and 0.88 over a 2-week period. Additionally, the Spanish version shows adequate test–retest reliability (ICC = 0.84)^51^, while the Cronbach’s alpha for the current sample is α = 0.90.

##### Self-schema

The Brief Core Schema Scale (BCSS) is a 24-item measure concerning beliefs (cognitive) about the self and others. For this research only the ‘positive beliefs about self’ section, i.e. the positive self-schema was utilized. This section includes six items, rated from 0 to 4 according to their degree of belief regarding statements like “I am good” and “I am successful”. Internal consistency (Cronbach’s alpha) of the original measure for positive self-schema in nonclinical samples and clinical samples are α = 0.78 and 0.86, respectively^52^. Internal consistency of the current sample is α = 0.69.

#### Transcendence

Transcendence is a measure that captures life goals and purpose, and is based on the spirituality, conformity and community dimensions of the Aspiration Index^41,53,54^. This self-report measure contains 12 items, ranked 1–9 based on the importance and likelihood of them taking place for the respondent. The original version has Cronbach’s alpha measures between 0.72 and 0.89, and in the current sample the internal consistency was α = 0.79 for importance and α = 0.80 for likelihood.

### Procedure

After obtaining ethical approval for cross-sectional research for a broader project about psychopathology, personality and coping strategies in adolescence by the Ethics Committee of the Universitat Autònoma de Barcelona (CEEAH number 2603, Spain) and in accordance with the Declaration of Helsinki, adolescent participants were recruited through willing secondary schools in Catalonia for a broader project encompassing adolescent personality, coping and mental health. Families were provided with detailed information regarding the study by means of a letter distributed through the schools, then invited to a meeting to resolve any concerns or questions before participating. Informed consent was received from parents before beginning data collection. Data were recruited in schools to simplify logistics. Participants received questionnaires in a sealed envelope with anonymous identity encryption using alphanumeric codes. Participants with missing or out-of-value data were later contacted to correct and finalize their participation.

### Statistical analysis

Data are available publicly available via supplementary materials. Because of the complexity of interpreting an interaction between two quantitative factors with potential opposite effects, we operationalized this paired effect using combinations of high or low attention to emotions with high or low emotional clarity. This yielded four profiles of emotional self-awareness which better allow to test differences between combinations. Dimensions of attention to emotions and emotional clarity are originally quantitative variables which, for the purposes of this research, were transformed into dichotomous variables (‘high’ vs. ‘low’) to create four defined profiles.

While dichotomizing variables is not standard practice, in the case of the dimensions of emotional self-awareness, this is sensible as the two opposite profiles could lead to very different mental health outcomes. In terms of continuous measures, an interaction between High Attention x Low Clarity (which, for example, could be operationalized scores of 20 and seven that would interact to equal 20 times seven, or 140) would yield the same score as the opposite interaction of Low Attention x High Clarity (seven times 20, also equal to 140). The difficulty of interpreting an interaction between two quantitative factors expected to show opposite effects was the main reason for the transition from the dimensional measures, difficult to combine, to a qualitative perspective based on ‘profiles’.

Thus, the cut-off for ‘high’ attention or clarity was selected at the second tercile (67th percentile) because it was considered this allowed to firmly capture those with ‘high’ attention or clarity while maintaining sufficient group sizes for analysis in all the four groups. According to dichotomization of the two variables, attention and clarity, with two levels each, high and low, four comparison groups were created: those with balanced high attention and high clarity (hereafter ‘*both high*’, referring to a sort of ‘full insight’), those with a negative imbalance based on high attention but low clarity (further referred to as ‘*high attention only*’), those with a positive imbalance based on low attention and high clarity (further referred to as *‘high clarity only*’), and those with balanced low attention and low clarity (hereafter ‘*neither*’, expressing ‘out of sight, out of mind’).

We conducted power analyses using Stata V. 17.0. With α = 0.05, power (1 − β) = 0.8 and four covariates (one explanatory variable, three control variables). The sample needed to detect a small-to-medium effect size of R^2^ = 0.07 was 164. All analyses exceeded this sample size. According to the study design, regression models were conducted instead of ANOVAs to ensure that it was possible to control for potential confounding variables in a cross-sectional study with no randomization among groups. Two regression models were conducted to answer research questions. The first one, using ‘*neither*’ as the reference group, explored the two hypotheses, predicting that (a) individuals with ‘*both high’* would demonstrate more favorable positive and negative mental health indicators compared to those with ‘*neither’* and (b) that those with *‘only high attention’* would demonstrate worse outcomes on all measures compared to the two aforementioned groups (‘*both high’* and *‘neither’*). The second regression model uses ‘*both high’* as the reference group to explore (by comparing between them) whether ‘*high clarity only’* differs from *‘both high’* in their contributions to mental health.

Both models met the assumptions of normality, linearity, homoscedasticity, and absence of multicollinearity. The regression models tested the effect of emotional self-awareness combinations against negative mental health indicators (anxiety and depression) and positive mental health indicators (resiliency, positive self-schema, self-esteem, and transcendence), controlling for age, sex and socioeconomic status (potential confounding variables).

All data analyses were conducted using IBM SPSS Statistics v20.0. We present the results of these associations as regression coefficients (*B*), with corresponding confidence intervals (95% CI) and *p* values (*p*).

## Results

### Descriptive results

Table 1 shows descriptive statistics and correlations for all variables of interest. All significant correlations showed the expected direction. Sex distribution amongst emotional self-awareness pairings includes 13.5% of boys in the ‘both’ group, 21.5% for clarity only, 19.2% for attention only and 45.8% for neither.

**Table 1:** Descriptives and correlations.

	All *n* = 264	Neither *n* = 121	Clarity only *n* = 56	Attention only *n* = 52	Both *n* = 35	1	2	3	4	5	6	7
	*M (SD)*					Pearson correlations *p < .05 **p < .001						
Resilience (*n* = 260)	26.5 (6.29)	26.0 (5.83)	27.8 (6.58)	24.7 (6.42)	29.0 (6.24)	–						
Self-Esteem (*n* = 260)	21.2 (5.48)	21.4 (4.53)	23.8 (4.50)	16.8 (6.03)	23.1 (5.25)	0.48**	–					
Self-Schema ( +) (*n* = 255)	13.7 (5.26)	12.8 (4.88)	15.6 (4.90)	12.1 (5.13)	15.7 (5.93)	0.36**	0.59**	–				
Transcendence (*n* = 166)	352.3 (128.02)	324.1 (120.33)	371.2 (139.23)	350.2 (123.13)	411.0 (125.00)	0.06	0.15	.12	–			
Depression (*n* = 261)	9.0 (8.23)	8.6 (7.61)	5.4 (5.40)	15.5 (9.76)	6.5 (6.00)	− 0.36**	− 0.68**	− 0.38**	− 0.07	–		
Anxiety (*n* = 258)	42.4 (15.00)	40.9 (14.49)	37.4 (14.37)	51.2 (14.66)	42.3 (13.16)	− 0.25**	− 0.49**	− 0.31**	0.11	0.43**	–	
Age	14.64 (1.72)	14.4 (1.71)	14.5 (1.61)	15.6 (1.57)	14.4 (1.69)	− 0.06	− 0.26**	− 0.08	− 0.04	0.28**	− 0.01	–
SES (*n* = 261)	39.25 (13.53)	40.4 (13.74)	41.1 (12.59)	35.3 (14.42)	38.2 (12.16)	− 0.04	0.12*	0.06	0.03	− 0.11	− 0.18**	− 0.07

### Regressions

Table 2 presents *B*, *p*, and 95% confidence intervals (CI) after conducting linear regression with a predictor which contained the four emotional self-awareness profiles. To explore whether the *both high* group had the best outcomes and the *neither* group had the worse outcomes, the group with *neither* was defined as the reference group. Indeed, the table shows that compared to the group with *neither*, the group with *both high* revealed a positive effect on three of the six indicators with the strongest effect sizes compared to the other two groups. Specifically, those with *both high* show significantly higher positive self-schema (*B* = 2.83, *p* = 0.004), more resiliency (*B* = 2.76, *p* = 0.015) and higher transcendence (*B* = 82.4*, p* = 0.003). In contrast, the effect of *high attention only* compared to *neither* was associated with lower self-esteem (*B* = − 3.38*, p* < 0.001) and more symptoms (*B* = 5.82, *p* < 0.001 for depression; *B* = 9.37, *p* < 0.001 for anxiety).

**Table 2:** Regression effects (B) of comparing three self-awareness profiles (attention + clarity, attention only, clarity only) with neither as reference group, controlling for age, sex and socioeconomic status.

	Neither (attention nor clarity) *n* = 121								
	Both (attention + clarity) *n* = 35			Attention only *n* = 52			Clarity only *n* = 56		
	*B*	*p*	95% CI	*B*	*p*	95% CI	*B*	*p*	95% CI
Resiliency (*n* = 260)	2.76	0.015	0.53, 4.99	− 1.67	0.119	− 3.76, 0.43	1.62	0.098	− 0.30, 3.53
Self-Esteem (*n* = 260)	1.73	0.059	− 0.06, 3.53	− **3.38**	**< 0.001**	− **5.06, **− **1.69**	**2.07**	**0.009**	**0.53, 3.61**
Positive Self-Schema (*n* = 255)	**2.83**	**0.004**	**0.92, 4.74**	− 0.17	0.848	− 1.95, 1.60	**2.45**	**0.003**	**0.82, 4.07**
Transcendence (*n* = 166)	**82.4**	**0.003**	**28.05, 136.75**	46.47	0.086	− 6.54–99.47	32.2	0.238	− 21.33, 85.80
Depression (n = 261)	− 2.25	0.106	− 4.97, 0.47	**5.82**	**< 0.001**	**3.26–8.38**	− **3.11**	**0.009**	− **0.77, 6.82**
Anxiety (n = 258)	1.10	0.685	− 4.23, 6.44	**9.37**	**< 0.001**	**4.42–14.33**	− 2.59	0.262	− 7.11, 1.94

Significant values are in [bold].

Beyond Table 2, while no significant effects were revealed for any indicator when comparing *high clarity only* to *both high*, *high attention only* was associated to an impairment on almost all indicators when compared to *both high*, with the exception of transcendence (for resiliency, *B* = − 4.42, *p* = 0.001, CI − 7.1, − 1.8; for self-esteem, *B* = − 5.11, *p* < 0.001, CI − 7.3, − 3.0; for positive self-schema, *B* = − 3.00, *p* = 0.009, CI − 5.3, − 0.7, for depression *B* = 8.07, *p* < 0.001, CI 4.8, 11.3; for anxiety, *B* = 8.27, *p* = 0.011, CI 1.92, 14.62). Although there was no difference between *both high* and *high clarity only* for any outcome, compared to the group with *neither*, *high clarity only* showed an effect in most cases that *both high* did not. Specifically, *high clarity only* but not *both high* showed a positive effect regarding depression (*B* = − 3.11, *p* = 0.009) and self-esteem (*B* = 2.07, *p* = 0.009) when compared with *neither* (Table 2).

**Figure 2:**
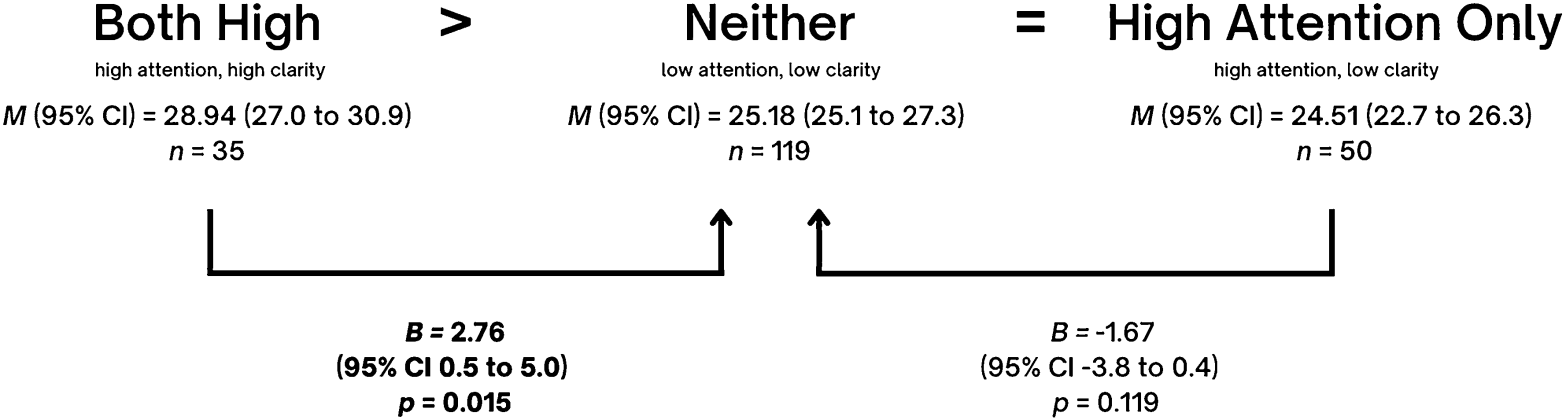
Effect sizes of High Attention Only (high attention, low clarity) and Both High (high attention, high clarity) on **Resilience,** using Neither (low attention, low clarity) as the reference category. Mean frequencies of resilience with self-awareness pairings and effect sizes of *Both High* and *High Attention Only* on resilience, using *Neither* as the reference category, and controlling for age, sex and socioeconomic status. Note that in the figure, ‘*Both High’* signifies the group with high attention and high clarity, ‘*Neither’* signifies the group with neither high attention nor high clarity, and ‘*High Attention Only*’ signifies the group with high attention but low clarity. Adjusted mean (*M*), linear regression coefficients (*B*), 95% mean confidence interval (95% CI) and *p* values (P) are indicated in the figure.

**Figure 3:**
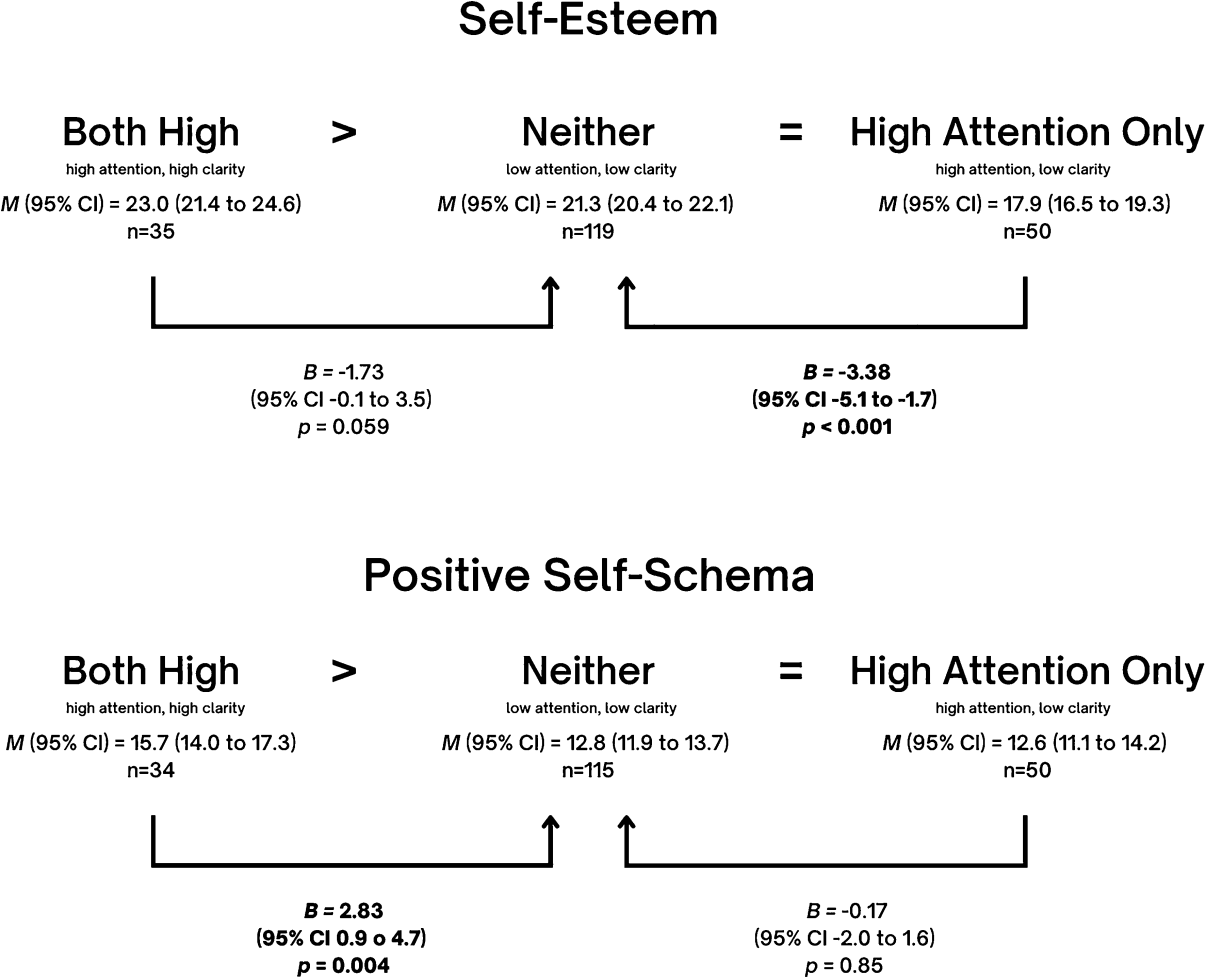
Effect sizes of High Attention Only (high attention, low clarity) and Both High (high attention, high clarity) on **Self-Image (Self-Esteem and Positive Self-Schema),** using Neither (low attention, low clarity) as the reference category. Mean frequencies of resilience with self-awareness pairings and effect sizes of *Both High* and *High Attention Only* on self-image (self-esteem and positive self-schema), using *Neither* as the reference category, and controlling for age, sex and socioeconomic status. Note that in the figure, ‘*Both High’* signifies the group with high attention and high clarity, ‘*Neither’* signifies the group with neither high attention nor high clarity, and ‘*High Attention Only*’ signifies the group with high attention but low clarity. Adjusted mean (*M*), linear regression coefficients (*B*), 95% mean confidence interval (95% CI) and *p* values (P) are indicated in the figure.

**Figure 4:**
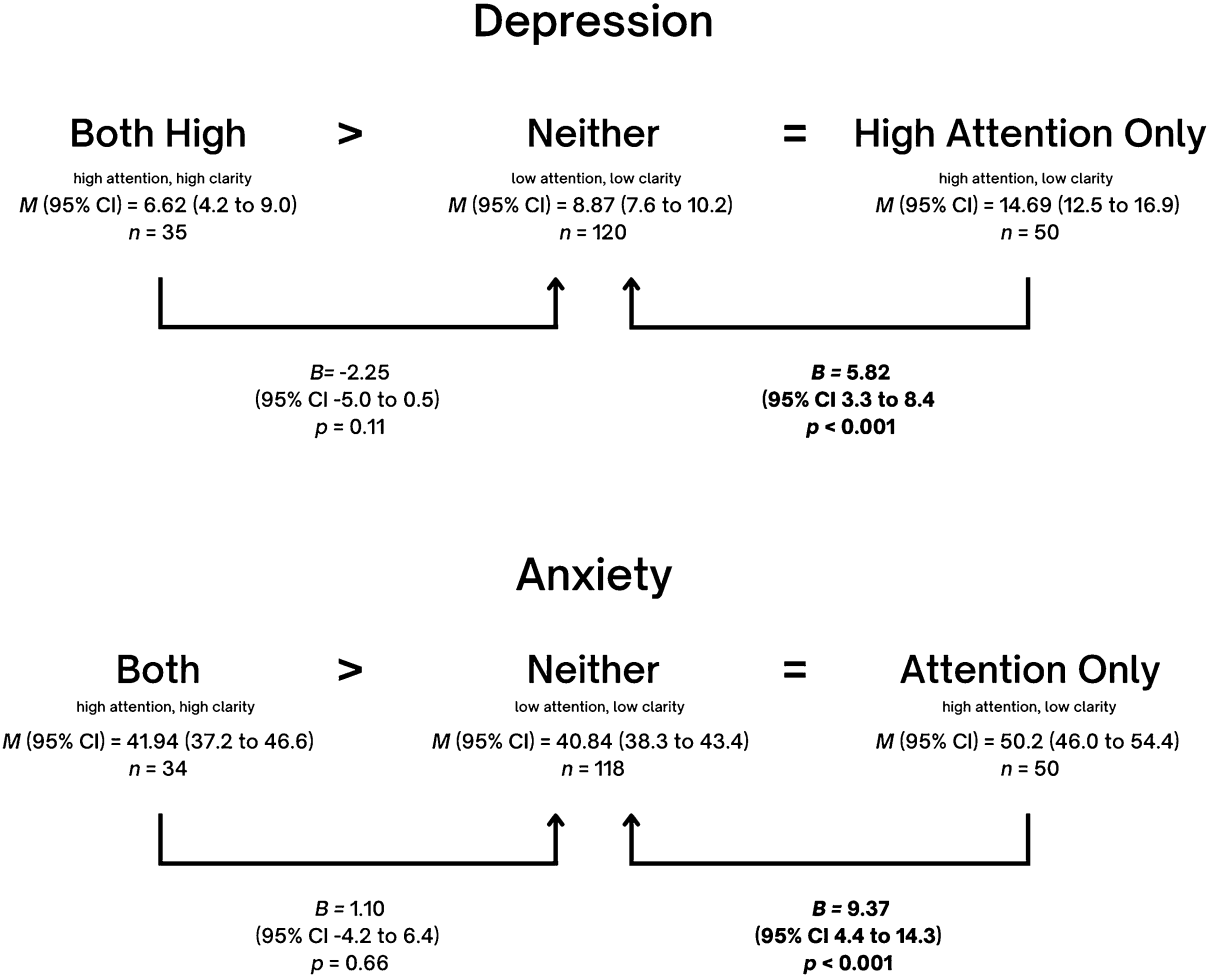
Effect sizes of High Attention Only (high attention, low clarity) and Both High (high attention, high clarity) on **Symptom Outcomes (Depression and Anxiety),** using Neither (low attention, low clarity) as the reference category. Mean frequencies of resilience with self-awareness pairings and effect sizes of *Both High* and *High Attention Only* on symptom outcomes (depression and anxiety), using *Neither* as the reference category, and controlling for age, sex and socioeconomic status. Note that in the figure, ‘*Both’* signifies the group with high attention and high clarity, ‘*Neither’* signifies the group with neither high attention nor high clarity, and ‘*High Attention Only*’ signifies the group with high attention but low clarity. Adjusted mean (*M*), linear regression coefficients (*B*), 95% mean confidence interval (95% CI) and *p* values (P) are indicated in the figure.

**Figure 5:**
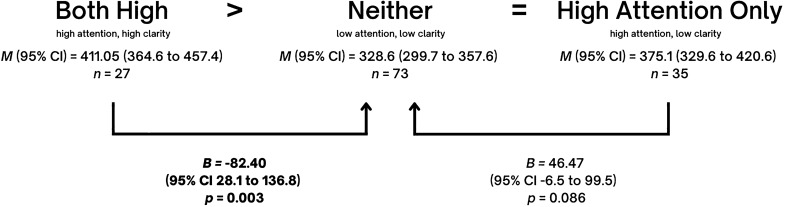
Effect sizes of High Attention Only (high attention, low clarity) and Both High (high attention, high clarity) on **Transcendence,** using Neither (low attention, low clarity) as the reference category. Mean frequencies of transcendence with self-awareness pairings and effect sizes of *Both High* and *High Attention Only* on resilience, using *Neither* as the reference category, and controlling for age, sex and socioeconomic status. Note that in the figure, ‘*Both High’* signifies the group with high attention and high clarity, ‘*Neither’* signifies the group with neither high attention nor high clarity, and ‘*High Attention Only*’ signifies the group with high attention but low clarity. Adjusted mean (*M*), linear regression coefficients (*B*), 95% mean confidence interval (95% CI) and *p* values (P) are indicated in the figure.

Detailed indications of hypothesis support for each mental health indicator are shown in Figs. 2, 3, 4 and 5. In all cases, our hypotheses were partially supported, either showing that *high attention only* is worse than *neither* in terms of depression, anxiety, and self-esteem, or showing that *both high* are better than *neither* in terms of positive self-image, resiliency or transcendence. One may note the intriguing result that full support of hypotheses was not found for any indicator.

## Discussion

The primary aim of this study was to elucidate the association between pairings of self-awareness (high/low attention with high/low clarity) and mental health, using indicators of both positive (resilience, positive self-schema, and transcendence) and negative (depression, anxiety) outcomes. We hypothesized that those with *both high* would show better outcomes on all measures for mental health, compared to those with *neither*. Further, we supposed that those with *high attention only* would show worse outcomes than those with *neither*, a hypothesis that was based upon previous research, which suggests a ‘dark side’ to emotional awareness for those with high levels of attention^30^.

Here we report partial support for our two hypotheses and discuss an interesting result regarding our exploratory analysis. Those with *both high*, compared to those with *neither*, demonstrate higher positive self-schema, resiliency and transcendence, but not higher self-esteem, lower depression or lower anxiety. Interestingly, the second hypothesis regarding *high attention only* compared to *neither* is supported for self-esteem, depression and anxiety outcomes, precisely on the outcomes that are insignificant for those with *both high*. These complementary results which partially support opposing predictions suggest the importance of clarity when attention is high, either improving positive indicators of mental health such as positive self-schema, resiliency or transcendence, or preventing impairment on depression, anxiety and self-esteem, which happens when attention is high but lacks clarity, but disappears when clarity is high as well. This is consistent with previous research that supports attention as the subfactor of emotional self-awareness susceptible to damaging mental health^55^, particularly when it is not combined with a balanced level of emotional clarity^24^. Further, it supports importance of clarity as a protective factor, thus logically affecting resiliency and transcendence, as we discovered, or attenuating the impact of high attention on symptoms.

In accordance with previous research, symptomatology (including low self-esteem) would be the expected result of a high, and especially imbalanced, level of attention to emotions. In daily life, this may be indicative of excessive awareness of feelings (i.e. ‘I am nervous!’) without further processing to integrate and understand emotions (i.e. ‘I am nervous because…’), rather than recognizing one’s emotions, applying the mental effort to understand them (clarity), and then moving on. Often referred to in mindfulness as being the ‘quiet observer’ or ‘peripheral observer’, this phenomenon consists of an ability to process and be aware of emotions (and indeed, all human experiences) from a place of detachment, allowing for deeper understanding and processing of emotions, which is likely aligned with emotional clarity. This suggestion is also consistent with previous research suggesting ‘overwhelm’ with the high attention pairing^56^. Without a balanced level of emotional clarity to calibrate the harmful effects of emotional attention alone, it seems sensical that one may find themself in a negative feedback loop for distressing or harmful thoughts, which over time, may exacerbate and result in common mental health symptoms.

Comparing the differences between those with *high clarity only* and those with *both high* was presented as an exploration to see whether there were, in fact, differences between those with what could be considered ‘implicit self-awareness,’ or low attention to their emotions but an almost ‘default’ understanding of them, and those with *both high*.

When comparing *high clarity only* with *both high*, no differences appeared regarding any indicator. Because the clarity dimension is high in both profiles, and this is the protective dimension as supported by the literature, the lack of difference could be attributed to the similar level of the ‘active ingredient’ (clarity) of the two profiles in this sense. However, when we compared both profiles (with and without attention) with *neither*, those with *high clarity only* showed a positive association with mental health in two of the three cases in which *both high* did not. Specifically, when compared with *neither*, *both high* showed no differences regarding depression, anxiety, or self-esteem, while *high only clarity*, in contrast, was associated with significantly lower depression and higher self-esteem than *neither* attention nor clarity.

This suggests that, when compared with *neither*, the protective effect of high clarity depends on which element of mental health is being evaluated. Apparently, for more cognitive indicators, which correspond with positive indicators of mental health (i.e., self-schema, resiliency, and transcendence), more explicit reflection may be more helpful since *both high* is associated with more positive self-schema, higher resilience and higher transcendence when compared with *neither*, while *high clarity only* does not show this positive effect. In contrast, for more affective dimensions (i.e., symptoms: depression, anxiety, (low) self-esteem), *high clarity only* without attention could be better, possibly providing the more automatic and rapid response required in presence of emotional distress. This is consistent with the dual-process theory^57^ which suggests that type 1 processing (automatic, contextualized, fast processing) is more efficient for evolutionary threats and is usually active when arousal is high, while type 2 processing (deliberate, reflective, slow processing), considered more productive and closer to normative standards, is the one contributing cognitive processes requiring reflection such as self-schema, ego-resiliency and transcendence.

When compared with the reference pairing *neither*, *high attention only* is harmful for symptomatology, but when clarity is introduced in tandem with attention, this impairment disappears. Then, when clarity remains and attention does not (*high clarity only*), symptomatology is, in fact, lower. This leads us to reflect on the imbalanced pairings, whereby one factor is high, and the other is low. *High clarity only* has beneficial associations with mental health indicators, while *high attention only* shows the poorest associations with mental health. Other studies suggest that imbalanced combinations of attention and clarity (e.g. one high, the other low) may provide opposite effects in the association between a risk factor and an outcome: while *high attention only* may strengthen the risk for psychopathology, clarity only appears to attenuate it. The current result further suggests that the effect of *high clarity only* (implicit awareness) could be less general than previously hypothesized, and in fact be beneficial for specific aspects of mental health (i.e. cognitive aspects) but not as much for affective ones.

A similar dynamic was revealed when analyzing results for self-esteem and self-schema, which, despite both falling under the umbrella of self-image, are positively associated with different pairings of attention and clarity. While self-schema was positively associated with *both high*, self-esteem was not and, in fact, self-esteem was negatively associated with *high attention only*, like the result with anxiety and depression. This result deserves further discussion.

Self-schema and self-esteem are neuroscientifically distinct^10^. As such, their associations with different combinations of self-awareness are not inconsistent nor entirely surprising. Self-schema indicates a more cognitive or controlled understanding of the self from outside (e.g. I am objectively successful) while self-esteem is indicative of how one affectively *feels* about the self from the *inside* (e.g. I don’t feel successful) and could be considered ‘affective’ and ‘automatic’. It is possible that complete emotional self-awareness (*both high*) favors a process considered ‘more cognitive’ (self-schema) but not necessarily more affective (self-esteem). In fact, some affective processes (self-esteem, depression) may benefit more from less attention than more clarity, which could explain the association of *high clarity only* with self-esteem, but *both high* with self-schema. Future studies with more thorough assessments of self-awareness dimensions should shed new light on these questions.

Like all research, this study cannot stand alone in its implications regarding the roles of attention and clarity of emotional self-awareness for mental health, as it does have some limitations. Most obviously, this research is cross-sectional. While some prior evidence suggests the developmental nature of attention to emotions and emotional clarity for developing or maintaining mental health, definitive judgment regarding the roles of these factors should only be made with substantial replication and ideally, longitudinal data. Further, though we chose to measure attention to emotions and emotional clarity using the TMMS-24 because this is the most widely utilized measure to assess this construct, the TMMS-24, along with the Levels of Emotional Awareness Scale^58^ are the only two ways to assess attention and clarity regarding internal emotional experiences, and better instruments are urgently required to improve self-awareness assessment so that it is on par with similar concepts for *others*, like Theory of Mind and social cognition. Finally, we recognize that different schools of thought in emotional awareness research may refer to overlapping or nearly identical constructs by different names^59^ and this could be a limitation of the present study.

Generally, our results supported that high levels of both attention to emotions and emotional clarity are beneficial in terms of positive mental health indicators, specifically self-schema, resiliency, and transcendence, and that those who have high attention to emotions but lack clarity to balance out this attention are typically worse off regarding symptomatology, showing more depression and anxiety symptoms. Given the adolescent, non-clinical sample, this knowledge has the potential to yield widespread effects when applied to community groups who have not yet experienced the onset of a mental health condition. In clinical practice, it may be particularly helpful to direct adolescents toward ‘knowing themselves,’ by paying attention to and fostering clarity regarding emotions, with the aim of eliciting lasting protective factors for mental health, all the while diminishing the negative ones.

In sum, the Ancient Greeks seem to have been correct–this investigation regarding the associations of emotional self-awareness pairings and mental health indicators shows value in ‘knowing thyself’. Nonetheless, findings suggest that ‘knowing thyself’ is a multidimensional concept; the benefits of knowing ourselves are specific to balanced versus imbalanced combinations of attention to emotions and clarity regarding them, and further depend on the aspects of mental health being evaluated.

While intervening before adolescents meet clinical criteria for mental health conditions is ideal, it is not always achieved. This new evidence stresses the importance of fostering emotional *clarity* no matter the stage of mental health symptoms, independent of the level of attention to emotions. This evidence suggests that in individuals with alexithymia, for example, strictly focusing therapeutic efforts on paying more attention to emotions would not yield as much success as focusing on emotional clarity, which is beneficial when paired with a high level of attention to emotions, and alone.

Further, considering the adolescent, non-clinical sample, this knowledge may have ripple effects when applied, particularly before the onset of psychopathology. Our findings provide new evidence for how pairings of attention to emotions and clarity regarding them could positively impact mental health. The protective effect of emotional clarity, sometimes paired with high attention (explicit and conscious emotional awareness), and others with low attention (implicit or automatic awareness) introduces new challenges in the training of the general, developmental population in this skill that is typically not the focus of child-rearing. While traditional cognitive skills like language and thought are thoroughly covered in public educational systems (i.e. before therapeutic intervention is strictly necessary), education on emotional self-knowledge falls short despite the 2000-year-old saying that we must ‘know thyself’. It is attractive to speculate that training new generations in emotional self-awareness or even self-knowledge in general could help to introduce natural resilience factors across development, thus leading to better socioemotional skills in subsequent generations. This could help to invert the cycle of emotional illiteracy that remains to be of much consequence in public education.

### Supplementary Information

Supplementary Information 1.

Supplementary Information 2.

## Data Availability

The data analyzed in this publication can be accessed as supplementary material.
