# Supplementary material for: Emotional self-knowledge profiles and relationships with mental health indicators support value in ‘knowing thyself’
Source: Sci Rep. 2024 Apr 4;14:7900. doi: 10.1038/s41598-024-57282-w (PMC10991446; doi:10.1038/s41598-024-57282-w)

**CONTROL VARIABLES**

**ASEXE**= Chromosomic sex (0=Male, 1=Female)

**Age.Year** = Age in years

**HH**= Hollingshead's Socio-Economic Index (higher value indicates a higher level)

**Age.Month** = age in months

**INDEPENDENT VARIABLES**

**TMS67.00**= Dummy variable using 00 as reference.

      Code:

      0 means low (< Percentile 67)

     1 means high (> Percentile 67)

      The first number refers to attention

      The second number refers to clarity

      Values:

   1 = 11 (high attention and high clarity) (both)

   2 =  01 (low attention and high clarity)

   3 = 10 (high attention and low clarity)

   4 = 00 (low attention and low clarity) (neither) (Reference category)

**TMS67.11** = Dummy variable using 11 as reference

Code:

0 means low (< 67^th^ percentile)

1 means high (> 67^th^ percentile)

The first number refers to attention

The second number refers to clarity

Values:

1 = 11 (high attention and high clarity) (both) (reference category)

   2 =  01 (low attention and high clarity)

   3 = 10 (high attention and low clarity)

   4 = 00 (low attention and low clarity) (neither)

**TMMS.att** = Trait Meta-Mood Scale - Attention scale (Sum of items 1 to 8): Dimensional

**TMMS.cla** = Trait Meta-Mood Scale - Clarity scale (Sum of items 9 to 16): Dimensional

**DEPENDENT VARIABLES**

**RESIL.CD** = Resilience, measured with the 10-item Connor Davidson Resilience Scale

**SE.ROSn** = Self-esteem, Rosenberg Self Esteem Scale

**SS.Pos** = Positive Self Schema, Brief Core Schema Scale

**BDIn** = Beck Depression Inventory (Total Score; A higher score indicates more depressive symptoms)

**MASCn** = Multidimensional Anxiety Scale for Children (Total Score; A higher score indicates more general anxiety)

**Transcen** = Transcendence, Aspiration Index

**TMMS 24 ITEMS**

**TMMS_01** to **TMMS_24** = Trait Meta-Mood Scale items 1 to 24. Scores range from 1 (Totally disagree) to 5 (Totally agree). Labels include the Spanish version, followed by the English version of each item. The English version of the items are pasted below.


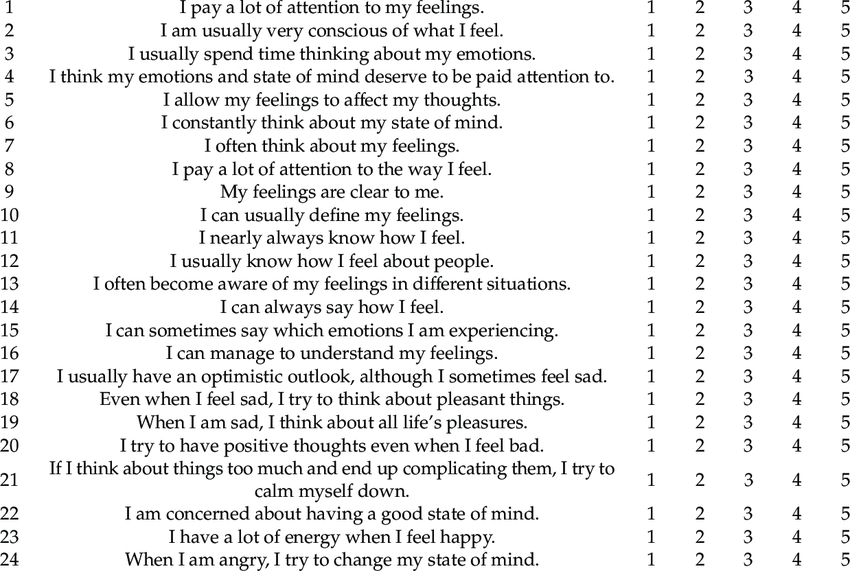

Supplement: Supplementary file 2 — Supplementary Information 2. [file 41598_2024_57282_MOESM2_ESM.docx]
